# One-Channel Wearable Mental Stress State Monitoring System

**DOI:** 10.3390/s24165373

**Published:** 2024-08-20

**Authors:** Lamis Abdul Kader, Fares Al-Shargie, Usman Tariq, Hasan Al-Nashash

**Affiliations:** 1 Biomedical Engineering Graduate Program, College of Engineering, American University of Sharjah, Sharjah P.O. Box 26666, United Arab Emirates; g00068616@aus.edu; 2 Department of Rehabilitation and Movement Sciences, Rutgers University, Newark, NJ 07107, USA; fares.yahya@rutgers.edu; 3 Department of Electrical Engineering, College of Engineering, American University of Sharjah, Sharjah P.O. Box 26666, United Arab Emirates; utariq@aus.edu

**Keywords:** mental stress, galvanic skin response (GSR), electroencephalography (EEG), wearable system, monitoring, stress detection, Stroop color and word test (SCWT), machine learning

## Abstract

Assessments of stress can be performed using physiological signals, such as electroencephalograms (EEGs) and galvanic skin response (GSR). Commercialized systems that are used to detect stress with EEGs require a controlled environment with many channels, which prohibits their daily use. Fortunately, there is a rise in the utilization of wearable devices for stress monitoring, offering more flexibility. In this paper, we developed a wearable monitoring system that integrates both EEGs and GSR. The novelty of our proposed device is that it only requires one channel to acquire both physiological signals. Through sensor fusion, we achieved an improved accuracy, lower cost, and improved ease of use. We tested the proposed system experimentally on twenty human subjects. We estimated the power spectrum of the EEG signals and utilized five machine learning classifiers to differentiate between two levels of mental stress. Furthermore, we investigated the optimum electrode location on the scalp when using only one channel. Our results demonstrate the system’s capability to classify two levels of mental stress with a maximum accuracy of 70.3% when using EEGs alone and 84.6% when using fused EEG and GSR data. This paper shows that stress detection is reliable using only one channel on the prefrontal and ventrolateral prefrontal regions of the brain.

## 1. Introduction

In today’s world, mental stress is a recurring serious condition that occurs when the human body responds to a demand for change. While routine stress does not pose a danger to life, it can still trigger a fight-or-flight response. This response is activated when the sympathetic nervous system (SNS) sends signals to release stress hormones through sympathetic nerve fibers. These hormones elicit physiological and psychological changes associated with the response. Therefore, when short-term stress persists for a longer period, it can have repercussions on the human nervous system. Stress has a significant impact on the structure and function of the brain at the cellular and subcellular levels. Various factors, including the duration and type of stressor, age, and gender [1], influence the impact of stress on brain organization and performance. The hippocampus, prefrontal cortex, and amygdala are the major areas of the brain most affected by stress since they contain abundant glucocorticoid receptors [2]. These receptors regulate physiological and behavioral responses under normal conditions and during stress, help maintain homeostasis, and facilitate long-term adaptations. 

Furthermore, mental stress can also lead to cardiovascular diseases, depression, and insomnia. Economically, mental stress can be a burden since there are reports that show a decrease in companies’ general performance due to the elevation in employee stress levels in the workplace [3]. Thus, the detection of mental stress and monitoring its elevation are fundamental for individual health and society’s welfare. 

The available methods to detect mental stress include subjective, behavioral, and physiological techniques. The subjective experience of detecting mental stress mainly involves interviews and questionnaires [4]. These methods are affected by individual bias and systematic errors. Moreover, demonstrated behavioral techniques for stress responses, such as body gestures and facial expressions, have limitations related to conscious observation. Physiologically, to enable adaptation to stress conditions, it is critical to provide muscles and nerve cells with adequate energy sources [4]. The brain and the spinal cord make up the central nervous system (CNS). Through it, the autonomic nervous system (ANS) controls the heart’s electrical activity, gland secretion, blood pressure, and respiration function, among others, to preserve homeostasis in the human body [4,5]. The ANS enables and controls the body’s reaction to external and internal stimuli. 

Evaluations of ANS activity can be performed by recording and analyzing several physiological variables, including heart rate, respiration rate (RespR), electrocardiograph (ECG) activity, galvanic skin response (GSR) [6,7], and skin temperature. Continuous monitoring of a person’s stress levels is vital for understanding and managing personal stress. Luckily, wireless devices exist to monitor these physiological indicators. Using these devices, people can carefully track variations in their vital signs to improve their health in daily life [7]. However, there is no preferable approach to measuring stress since all these biological signals are affected by several factors, including skin diseases, humidity, environmental temperature, and other systematic errors [7]. 

Neuroimaging techniques provide a significant method to estimate neurophysiological activity accompanying stress triggers [7,8]. One of the most common techniques for stress assessments is employing electroencephalograms (EEGs). They reflect the brain’s electrical activity in real time in milliseconds, providing a high temporal resolution [8]. Furthermore, the prefrontal cortex is responsible for a large proportion of emotional processing [8]. Stress usually causes negative moods, such as depression and anxiety, resulting in a decrease in right prefrontal activity [8]. Thus, analyses of the frequency band powers delta (0.5–4 Hz), theta (4–8 Hz), alpha (8–13 Hz), and beta (14–30 Hz) in EEGs measured at the prefrontal cortex have been generally applied in previous stress studies [8,9,10,11]. Usually, alpha and theta are more significant frequency bands, as stress is mainly identified through the changes between them. In a state of stress, alpha decreases and theta increases, while in a relaxed state or state without activity, alpha increases and theta decreases [8,9,10,11,12,13,14].

Nonetheless, EEGs also have some disadvantages, such as the low spatial resolution on the scalp, the poor detection of neural activity below the upper cortex layers, the need for a controlled environment to use commercialized EEG systems and run experiments, and the inability to detect the exact locations at which different neurotransmitters are found [12,13,14]. As a result, the simultaneous analysis of both EEGs and another physiological signal like GSR is likely to provide a more precise assessment of stress [15,16,17,18,19,20], and one of the main objectives of this study is to verify this hypothesis. 

Skin conductance (SC) will be used along with EEGs for optimal stress assessment. SC is a method of determining the electrical conductance of the skin, in which its value can vary with the moisture level of the induced sweat. The electrical conductance of the skin can be used to measure the electrodermal activity (EDA) as a factor of emotional responses such as stress [21]. Sweat is controlled by the SNS, and the conductance of the skin is used as an indication of psychological or physiological arousal [22]. If the sympathetic branch of the autonomic nervous system is strongly aroused, the activity of the sweat glands also increases, which increases the conductance of the skin [22,23,24]. In wearable devices, this activity will be extracted through suitably arranged electrodes and electronic circuits for detecting and shaping the resulting signal known as GSR. 

GSR is used as a sign of physiological arousal. It measures the electrical resistance between two points and is basically a kind of ohmmeter. By applying a constant ac current between two sites on the skin, it is possible to observe a voltage drop through it. Consequently, it allows for the monitoring of the change in impedance for the GSR [18]. Emotions such as stress, excitement, and shock can produce fluctuations in GSR. Although several possible signals have been considered to detect stress, GSR has been considered as a measure of physiological and mental stress in many previous studies [24,25,26,27,28,29,30,31,32]. It has been reported that a higher GSR may be noticed for higher levels of stress [33]. Thus, GSR may be a relatively good indicator of stress [33,34,35,36,37,38,39,40]. However, the determination of the specific level of GSR for specific emotions is difficult. All emotions, like anger, fear, the startle response, sexual feelings, and the orienting response, may produce similar GSRs [30]. Hence, GSR alone may not be a sufficient indicator of stress, and using an additional biomarker would improve detection performance. Ultimately, for optimal mental stress detection, this paper will utilize EEGs and GSR along with subjective and behavioral information for the optimal monitoring of stress.

There are numerous tests used by researchers worldwide to induce stress in participants. These tests include the Trier social stress test (TSST), Stroop color and word test (SCWT), cold pressure test, and more. The SCWT involves reading out the color of the word when the color of the word is printed in a different color than what it represents. The SCWT induces stress through a contradiction between visual and linguistic perception. The SCWT has a high reliability and stability in measuring stress [40,41,42,43,44]. 

In terms of accomplishing continuous stress detection and monitoring in the workplace, device usability is key. For EEG systems, many clinical studies have utilized EEG channels following the 10–20 electrode system on hair-bearing scalp areas. This method can be tedious and inconvenient as it requires the use of a conductive electrode gel and a suitable preparation procedure [33,34]. Therefore, EEG recordings from hairless regions such as the forehead, or behind the ear, would be more suitable for a wearable monitoring system in the workplace [35,36]. For GSR or electrocardiogram (ECG) measurements from two or more electrodes, areas on the wrist, the palmar surface of the hands, fingers, plantar sites, or the chest are commonly used [36,37]. However, based on previous studies, it is currently necessary to use two different devices for simultaneous EEG and GSR recordings [28,29,30,31,32,33,34,35,36,37,38]. 

For the real-time monitoring of stress, various wearable commercial devices are available on the market. Some researchers have used these devices for data collection and the continuous monitoring of the stress levels of users. Others have developed their own wearable devices made from low-cost sensors. Each sensor is independent in terms of the acquisition of one physiological signal for the detection of stress [28,29]. This paper proposes a long-term monitoring system for the detection of stress through the integration of information from GSR and EEGs using one sensor. This is ideal for a workplace environment. In summary, this paper contributes the following to the literature:

- The development of a wearable system that integrates the information of EEGs and GSR, which can be used for the detection of elevations in stress. Usually, GSR systems are independent of EEGs and are used in research lab environments. Integrating both in a wearable system reduces the size of the device and the level of complexity while still maintaining a high accuracy.
- An EEG system comprising a 10–20 electrode system that uses the standard placement of electrodes on the scalp. They are high-density for use in research labs and clinical settings. Hence, it is difficult to use one-channel systems to identify and localize the source of stress. This study investigates and proposes a practical location for one-channel electrodes suitable for the detection of stress elevations using EEGs and GSR.
- This paper also proposes a frequency to induce sweat on the scalp and aid in the detection of stress using GSR.

All acronyms, full names, and descriptions in this manuscript are listed in the Appendix A.

## 2. Methodology 

### 2.1. Data Acquisiton System

**Figure 1:**
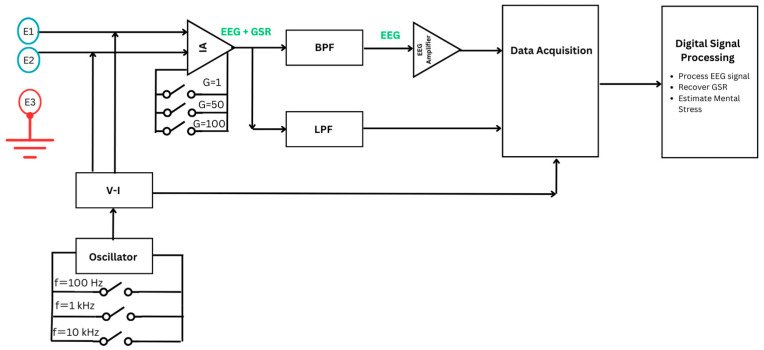
Block diagram of the hardware system designed for mental state monitoring.

The data acquisition system developed for mental state monitoring is illustrated in Figure 1 through a detailed block diagram. Within this diagram, a single channel is employed to capture both EEG and GSR signals. This dual-signal detection is achieved by utilizing three electrodes: positive, negative, and reference. Moreover, the position of the electrodes was investigated by using an innovative mechanical framework to attach the electrode at different areas of the subject’s scalp, namely, the prefrontal cortex (Fp1/Fp2) and ventrolateral frontal cortex (F7/F8), following the 10–20 electrode system [41]. 

Once the electrodes are positioned at the desired scalp location, EEG signals are recorded. However, to detect the GSR, a constant ac current at a specific frequency and magnitude is injected between the positive and negative electrodes to detect variations in the impedance. The variations in impedance occur when stress is induced and more sweat forms, which are detected as physiological changes in the subject’s mental state. This variation is detected with a voltage drop by the ac current signal applied to the skin [42,43]. As a result, the influence of stress through sweat and changes in GSR are integrated with the ac signal through amplitude modulation. The signal is then rectified, and envelope detection is utilized to separate the GSR signal from the carrier [44]. Based on the previous literature, the value of ac current injected into the skin is selected to be 10 μA [44,45,46,47,48,49,50]. Moreover, some studies used low frequencies for the carrier signal, such as 10 Hz, 70 Hz, and 100 Hz. Others use very high frequencies, such as 10–60 kHz, in portable GSR devices. To investigate this further, different frequency values, namely 100 Hz, 1 kHz, and 10 kHz, are used for carrying GSR information. The electrodes positioned at FP1 and FP2 are used as a pair to form a single differential channel for measuring both GSR and EEG signals. The EEG signal is captured by measuring the voltage difference between these two sites. This differential signal is then amplified using a differential amplifier, which enhances the signal quality by reducing common-mode noise.

The chosen frequency is a trade-off between the ability of the monitoring system to detect quick changes (which requires high frequency) and sensitivity for sweat duct activity (which requires low frequency). A low frequency may be a good choice in this respect [51]. However, Das et al. suggested that frequencies less than 100 Hz can damage and cause slight burning to the skin [28]. Thus, the carrier signal’s frequencies are considered to be higher than 100 Hz [28]. Furthermore, other studies suggest that frequencies less than 1 kHz can have the disadvantage of stimulating superficial nerves leading to complications. Many commercial GSR devices are set at relatively low frequencies. On the contrary, it should be noted that Nordbotten et al. found that measurements at higher frequencies used for obtaining SC levels do not provide estimations that are highly accurate [27,28]. 

Additionally, an instrumentation amplifier is used to amplify the voltage signals detected by the electrodes. The output of the instrumentation amplifier consists of a combined EEG and GSR-modulated signal. To separate both signals, a band-pass filter (BPF) for the EEG signal between 0.5 and 40 Hz was utilized. Also, a low-pass filter (LPF) with three cut-off frequencies was used to separate the carrier signal carrying GSR from EEG. Band-pass filtering and demodulation are performed using digital signal processing to reduce the size and cost of the wearable device. Digital signal processing includes cleaning the EEG, restoring the GSR through envelope detection, and using classifiers for EEG alone and for data fusion of both GSR and EEG to estimate the elevation in mental stress. 

**Figure 2:**
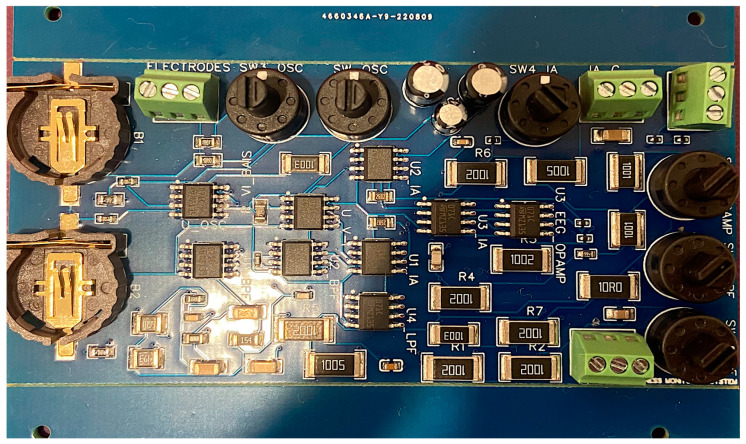
Fabricated PCB board of the data acquisition system for EEG and GSR.

The hardware of the data acquisition unit includes an oscillator circuit that offers three different frequencies: 100 Hz, 1 kHz, and 10 kHz; a voltage-to-current converter that injects a constant current to detect activity in sweat and carry GSR information; an instrumentation amplifier (IA); an EEG BPF from 0.5 Hz to 40 Hz; a GSR low-pass filter at three frequencies; and another EEG amplifier before the data acquisition stage. The data acquisition system was fabricated into a PCB of four layers. The bottom layer is the reference layer. The third inner layer is responsible for the dc power connections. Lastly, the two top layers are used for component connections. The PCB has a total of 69 components. The dc power (±3 V) is utilized from two batteries (CR2477). Figure 2 shows the fabricated PCB for data acquisition of both EEG and GSR.

The total production cost of the device is under USD 670. Specifically, we spent USD 50 on the printed circuit board, USD 20 on the bill of materials, and USD 50 on electronic manufacturing. The remaining USD 550 was spent on the mechanical framework, in which the wet electrodes are attached to the scalp and which allows for flexible electrode placement to examine various brain regions. This cost covers both the design in AutoCAD and the production of prototypes and the final version via 3D printing.

Although the electronics are relatively inexpensive, the overall cost is elevated by the mechanical framework, which is made from PA12—a material chosen for its balance of rigidity and flexibility. This added expense means that the device is not the cheapest available on the market. However, our investment in its mechanical framework enhances levels of comfort and usability, contributing to the device’s effectiveness as a wearable EEG system.

In summary, while the device is cost-effective in terms of its electronic components, it is not the cheapest wearable EEG device globally due to the significant cost of the mechanical framework.

Physiological data recording and analog signal processing were performed in real time. The demodulation of both physiological signals (EEG and GSR) was also implemented in real time. However, since there is a need to evaluate different classification models, the classification stage was implemented offline.

### 2.2. Mechanical Framework for Electrode

**Figure 3:**
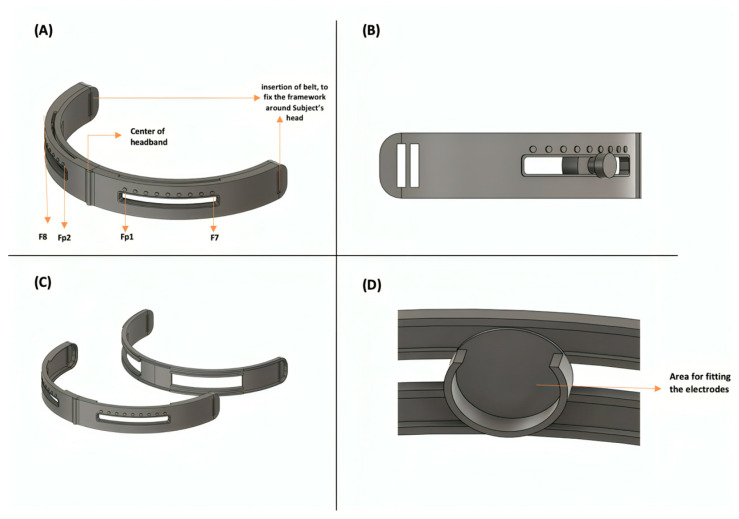
(**A**) Electrode positioning on Fp1 and Fp2 or F8 and F7. (**B**) Cursor/handle of the rail to move the electrode right and left. (**C**) Mechanical framework is divided into front and back parts. (**D**) Area used to fit the electrodes from behind for secure positioning of the electrodes at the desired location.

The mechanical framework is a headband used during the experiment for proper electrode placement on the head using a single channel, which means it uses two differential electrodes (positive and negative) and a reference electrode. The electrode locations designated on the headband refer to the desired locations that will be inspected for higher sensitivity in detecting elevated mental stress. These two locations are mainly on the forehead: Fp1 and Fp2, or F7 and F8. The reference electrode is located behind the right ear, at the Mastoid bone. Fp1 and Fp2 are measured on the left and right sides of the head by taking 10% of the measured head circumference from the nasion to the inion [52]. Similarly, for F7 and F8, 20% of the measured head circumference is taken from where Fp1 and Fp2 are in accordance with the 10/20 electrode placement [52]. The average distance between the nasion and inion for humans is about 32 cm [53]. Therefore, the overall circumference of this mechanical framework is 32 cm [53]. The design of the mechanical framework is shown in Figure 3A. The headband consists of a rail-like structure that allows the electrodes to slide from one location to another. The rail starts at location Fp1/Fp2, which is 3.2 cm from the center of the headband. In addition, the rail ends at F7/F8, corresponding to 6.4 cm. The electrodes are smoothly relocated from the start to the end of the rail using a cursor or handle at the front, as shown in Figure 3B. There is a secure fitting area for the electrodes attached to the handle at the back, as shown in Figure 3D. Thus, when the handle is moved across the rail to change position, the electrodes are also moved. Moreover, the framework is divided into two parts, front and back, that can be attached together. The front and back parts are shown in Figure 3C. Initially, both parts are detached, and electrodes are inserted at the desired positions indicated by the steps marked on top of the rail. Then, once both parts are attached, a narrow and hollow line at the top of the headband allows the electrode wires to move up, towards the hair and scalp. Thus, electrode wires encompass the subject’s head rather than their face and eyes to avoid discomfort during the experiments. The mechanical framework was printed using selective laser sintering (SLS) technology using PA12 polymerase, also called Nylon 12. This material is known to simultaneously be rigid and sturdy. Yet, it still holds flexible properties to properly position the device around the subject’s head during the experiment.

### 2.3. Mental Stress Task

The Stroop color and word task (SCWT) is a well-known cognitive test that we have adapted to induce psychological stress. In our protocol, we enhanced the stress-inducing properties of the SCWT through time constraints, negative feedback, and social evaluation threat. The SCWT in this study involved six color words presented in random order, with the printed word displayed in a color different from its meaning. Participants were required to choose the ink color rather than read the word. 

**Figure 4:**
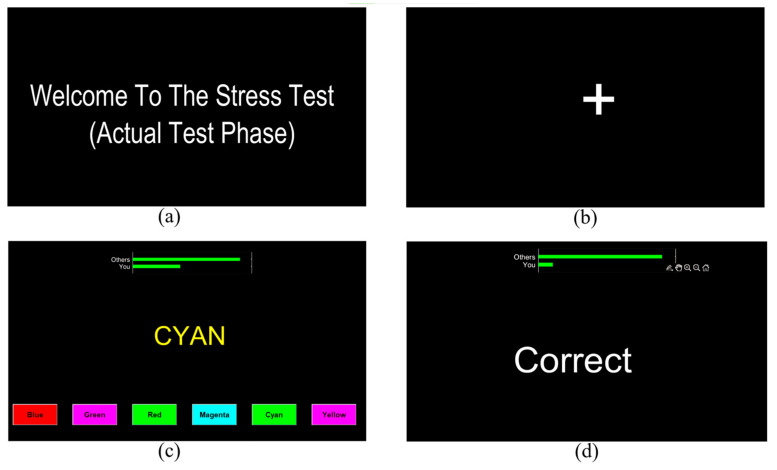
Stroop color and word task. (**a**) Instructions, (**b**) Resting period, (**c**) Stroop stimulus, (**d**) Trial feedback.

Figure 4 illustrates the SCWT interface used in the experiment. Each trial presented a single word with a random background color and six colored answer buttons. Participants had to respond quickly by left-clicking the correct answer. Feedback was provided after each trial, including “Correct”, “Incorrect”, or “Time’s up” messages. A performance indicator continuously displayed the participant’s standing relative to the fictitious “high-performing group”. This modified SCWT protocol combines cognitive interference, time pressure, negative feedback, and social evaluation to create a potent stress-inducing task. The full details of this stressor can be found in our previous studies [45,46], with the added stress-inducing elements enhancing its effectiveness in eliciting a measurable stress response.

### 2.4. Experimental Protocol

The experimental protocol is divided into two main phases: control and stress phase. The experimental setup consists of the hardware system shown in the block diagram of Figure 1 to acquire the physiological signals, the mechanical framework for placing the electrodes, and two computers. The first computer is used by subjects to take SCWT, coded on MATLAB R2021b. The second one is used for LabVIEW Student Software Suite Spring 2020, a software utilized to apply preliminary filtering of EEG and separation between the carrier signal and GSR signal by implementing demodulation through envelope detection. In addition, LabVIEW allows us to check and examine the signals visually in real time while the subjects are taking the SCWT. 

Initially, to ensure proper recording of EEG, the subjects are asked to close and open their eyes. These corresponding actions affect the alpha band, shown on the obtained signals from LabVIEW [47]. Then, the subjects are asked when they had caffeine and whether they smoked. A brief explanation is mentioned at the beginning of the experiment to inform the subjects of what is expected, such as minimizing movement while and the duration of taking the SCWT in each phase to reduce the noise of the recorded physiological signals. Then, the first part of the experiment starts, which is training. Training consists of explaining how the SCWT works by providing guidelines on how to select the correct answer. Furthermore, in training, the subjects are given 10 s to answer questions for 5 min. This time is sufficient for explaining the test and for subjects to score the correct answer during training. Once the subjects feel confident about SCWT, the control phase is established. The control phase is also carried on for five minutes but while acquiring the EEG and GSR signals. However, subjects are given only 4 s for every question. At the end of the control phase, some behavioral information is provided about the performance of every subject. Two types of data are given: the accuracy of the subject’s performance, or how many questions they got right, and the average time the subject took to answer each question. Such data are considered vital behavioral information for further assessment in the elevation of stress.

Then, the stress phase is executed by applying a stimulus for inducing physiological changes as stress via SCWT. In the stress phase, information about the average time taken to answer every question in the control phase is taken. Then, 80% of that time is calculated and is assigned as the overall time for choosing the correct answer in every question. Usually, this time varies between 1.2 and 2 s. In this case, stress is induced in subjects due to insufficient and short amount of time to complete every question. Also, between stress and control phase, a questionnaire is conducted. The questionnaire accounts for physiological information for measuring stress in each individual subject at each phase. This questionnaire is called the perceived stress scale (PSS). PSS is a standard stress evaluation instrument that examines how different situations can affect a subject’s feelings, thus measuring stress. Subjects are asked to answer this questionnaire quickly to indicate their direct feelings. PSS is made of 10 questions. Each question should be answered on a scale ranging from 0 to 4, in which never is indicated by (0) and very often is indicated by (4). When the summation of answers for each question is calculated, the inclusive PSS score ranges from 0 to 40. A range of 0–13 indicates low stress of the subject. Also, 14–26 implies moderate stress, and 27–40 signifies high stress [48,49,50]. 

**Figure 5:**
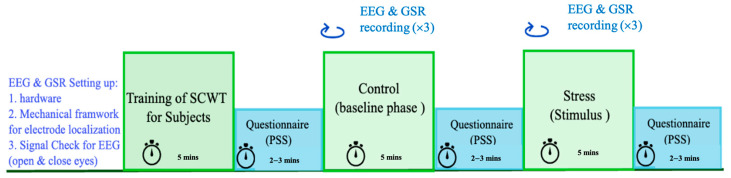
Experimental protocol for control and stress using SCWT.

EEG and GSR are simultaneously and continuously recorded during the control and stress phases. Moreover, randomization between the order of stress and control phases occurs for 50% of the subjects. Meaning in some subjects, the stress phase starts first, then control phase proceeds. This action will further increase the validation of the system and its sensitivity for detecting stress accurately. Additionally, the control and stress phases are implemented and recorded three times; each time, a different frequency is applied for the carrier signal. The purpose of this stage is to further examine which frequency of the ac excitation current will correspond to higher sensitivity of physiological changes in the GSR signal. The frequency selection during the experiment is randomized. Some subjects start from low first (100 Hz, 1 kHz, and 10 kHz), medium (1 kHz, 10 kHz, and 100 Hz), or high (10 kHz, 1 kHz, and 100 Hz) for further simultaneous recording of their GSRs. Consequently, this ensures that this research will recommend the best frequency, regardless of the level of accumulative overwork and fatigue in the subject. If the frequencies are not randomized (for instance, executing 10 kHz of the ac signal at the end of the experiment), more physiological changes might be shown at that frequency in GSR due to fatigue. Here, randomization is essential to avoid bias. Figure 5 shows a summary of the different phases of the experimental protocol and the duration to finish each phase. Ultimately, the overall duration of the experiment is 50–55 min.

### 2.5. Subjects

Twenty healthy right-handed adults participated in this experiment. These participants are students studying at the American University of Sharjah. The inclusion criteria for students to participate included those with normal color vision, without psychiatric or neurological disorders, who do not take long-term medications, and who do not show symptoms of drug addiction. Moreover, subjects should avoid drinking caffeine, energy drinks, and alcohol for at least 12 h before the task. The experimental protocol was approved by the university’s institutional review board (IRB). Ultimately, all subjects signed the consent form prior to conducting the experiment.

### 2.6. Data Analysis 

The data collected from the twenty subjects during the experiments were analyzed for EEGs and GSR. For GSR signals, we used statistical analysis to compare levels of sensitivity, accuracy, and p-test scores at each carrier frequency. As for EEG signals, the signals are pre-processed by splitting the noisy parts of each epoch signal that are full of artifacts. Then, the signal is initially band-pass filtered to obtain alpha, beta, theta, and delta bands for further analysis using frequency domain analysis. Gamma waves were not utilized in this paper since they are high-frequency brainwaves typically ranging from 30 to 100 Hz and are associated with higher cognitive functions, such as attention and problem solving. Moreover, they are often weaker and more difficult to detect accurately compared to lower-frequency bands like alpha, beta, and theta waves. In a single-channel setup, in which signal quality and noise management are already challenging, capturing and interpreting gamma waves can be particularly problematic [5]. 

In addition, lower-frequency bands such as alpha (8–12 Hz) and beta (13–30 Hz) have been more commonly associated with stress and emotional states in research. Furthermore, we should acknowledge the theta role in single-channel measurement. For further comparison, gamma waves are known for their role in complex cognitive functions and sensory processing, but their relationship with stress is not as straightforward as with other bands. The interpretation of gamma waves can be more complex and may require more advanced processing and analysis than is feasible with a single-channel device.

To proceed with data analysis for the aforementioned bands, features are extracted from the bands using power spectral density (PSD), and 10-fold cross-validation is used to differentiate between control and stress subjects. 

### 2.7. Signal Processing 

To obtain the GSR, an analog-to-digital converter (ADC) was used to digitize the 100 Hz, 1 kHz, and 10 kHz. The ac excitation current was separated from GSR on LabVIEW for initial signal processing. Envelope detection was used for the separation of integrated information. It involves using a band-pass filter to limit the excitation current signal (at different frequencies) to 5% of bandwidth (at the center frequency of 100 Hz, 1 kHz, or 10 kHz) since GSR ranges from 0 to 5 Hz. Then, an absolute value was utilized to rectify the signal. Furthermore, low-pass filter was used to extract the message. The low-pass filter’s cut-off frequency was twice the message’s frequency (GSR signal). Tone measurements help measure the frequency and amplitude of the displayed signal on either a waveform chart or waveform graph. As for EEGs, although the signal was acquired from a third order band-pass filter with a passband from 0.5 to 40 Hz, further filtering was applied on LabVIEW to avoid 50 Hz interference and other motion artifacts. 

### 2.8. Feature Extraction 

The processing of the EEG signals includes filtering, storing, and amplification [51]. The data acquisition system was used to analyze the EEG signal and extract its features. The EEG features can be divided into temporal and frequency features [20,52]. In this study, the wearable long-term monitoring device used frequency features. The EEG signal has five frequency bands: delta, theta, alpha, beta, and gamma. Frequency analysis was based on amplitude spectra provided by the Fourier transform [52,53,54]. Furthermore, the power spectrum is associated with the mental/physiological conditions of the subject. The power spectral density for a discrete signal X(t) that contains N samples is estimated using methods described in [29,53]. Subsequently, we used the calculated power spectrum features for classification. We estimated the channel-averaged power spectral density (PSD) in delta (1–4 Hz), theta (4–8 Hz), alpha (8–13 Hz), beta (13–25 Hz), and gamma (25–45 Hz) bands [53,54]. After PSD, a binary classifier between EEG frequency bands was used to determine two classifications of mental stress level: stressed or not stressed. Various machine learning algorithms were utilized in this work to compare their performance in distinguishing between two levels of stress, whether under mental stress or not. These machine learning algorithms include K-nearest neighbor (KNN), linear support vector machine (SVM), decision tree (DT), linear discriminate analysis classifier (DA), and naïve Bayes (NB) [55]. For each classifier, the data was randomly shuffled and divided into ten parts for cross-validation. One part was used for testing and the other nine were used for training the classifier. This process was repeated ten times until each of the ten parts were used in both testing and training. More details about the tuning parameters of the five classifiers can be found in our previous studies [54,55,56]. 

## 3. Results

This section presents the results from the testing and validation of the data acquisition system designed to monitor stress using EEGs and GSR. Furthermore, the classification of EEGs through the alpha, beta, theta, and delta between the control and stress phases is investigated using different machine learning algorithms. In addition, the optimum electrode location for EEGs, which was investigated between two positions on the frontal lobe, is examined through a statistical analysis (*p*-value) of EEGs. And finally, the effect of GSR at 100 Hz, 1 kHz, and 10 kHz carrier frequencies is investigated using machine learning. Ultimately, the results present the EEG and GSR accuracy, along with the recommended best frequency to measure GSR. 

### 3.1. Hardware Testing of the Monitoring System to Detect Stress

The EEG signal was modeled as a current source to test the acquisition of the hardware system. The current source was designed with 1 μA at 40 Hz. The EEG was modeled as a constant current source rather than a voltage source to show the integration of the system’s physiological signals, since the GSR measurement is carried by a constant ac current signal. The output of the instrumentation amplifier shows the modulated signal in which a 40 Hz wave is carried on the high frequency of the carrier signal. The amplitude of the modulated signal is accounted for by 1.4 V from the carrier signal (V-I converter) and 1.3 V from the EEG constant current source. 

**Figure 6:**
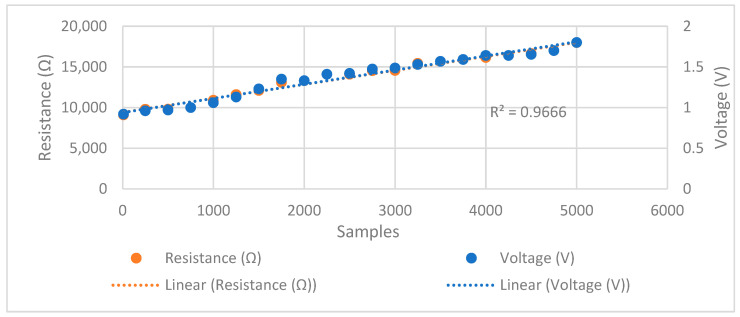
Validation of the sensitivity of the wearable system for changes in resistance and voltage of the GSR at constant current of 10 μA.

To further test the system, a potentiometer was placed at the position of the electrodes in Figure 1 (the block diagram) to demonstrate the potential changes in the skin’s electrical activity. As the resistance increases by twisting the knob on the potentiometer, a similar corresponding behavior was seen in the voltage of the demodulated signal, which models the GSR signal. The increase in resistance and voltage of GSR is modeled in Figure 6. This shows that the current will be constant despite variation in the resistance of the GSR. Thus, the proposed system is successfully tested for its performance. The circuit is calibrated to test for the accuracy in determining the change in skin impedance. The accuracy is measured against known resistances in the range of 1 kΩ to 20 kΩ. The change in resistance is directly related to electrical changes, such as the voltage drop produced by sweat and stimuli when stress is induced.

### 3.2. Removal of Noise and Artifacts from EEG 

To remove noisy artifacts that could be the result of the movement of the electrode, muscle contractions, the electromyogram (EMG), or the movement of the subject’s body during the experiment, a MATLAB code was executed to remove any EEG epoch that is above 0.15 V and below −0.15 V. The thresholding code was implemented for all 86 epochs of one EEG signal belonging to every subject. Although this code is useful in removing large signals of noise and artifacts, it does not get rid of eye blinks and electrocardiograms (ECGs) for some of the subject’s signals. The removal of eye blinks could help in increasing the accuracy of classification between the two levels of stress. Figure 6 validates the sensitivity of the wearable system for changes in resistance (orange) and voltage (blue) of the GSR at a constant current of 10 μA. 

### 3.3. Mental Stress Detection Using EEG 

For mental stress detection using EEGs, the raw EEG signals were used as input in the threshold code discussed above. Then, the EEG is segmented into epochs by using a window of size 1000. Hence, the total number of epochs used for each of the 20 subjects is 86 in each condition, whether it is in the stress or the control phase. The band power for every epoch was computed by separating the EEG into its frequency bands. Then, PSD is used on each band. Hence, the EEG has four extracted features, namely, the band power of delta, alpha, beta, and theta. Then the data were squeezed, and the mean of the band powers of every epoch in the EEG band was calculated. The output was one band power value in each epoch. This process is vital for arranging features and labeling them as stress or control features.

**Figure 7:**
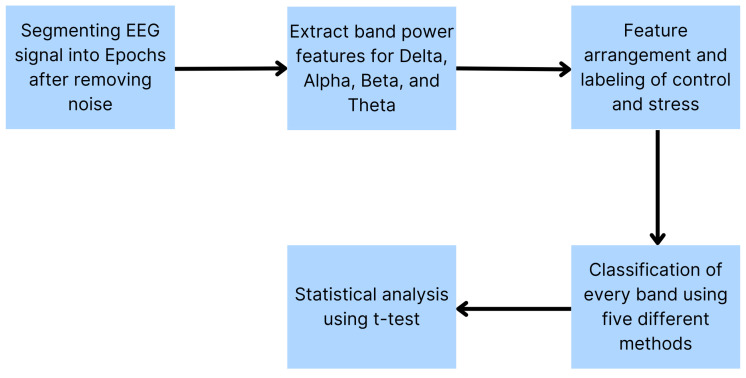
Framework of method used for EEG analysis and stress detection.

For feature arrangement, the data files of every subject are divided and arranged into control and stress feature matrices. The size of each matrix is 20 × 86: 86 epochs for all 20 subjects in each phase. Moreover, the control and stress feature matrix is normalized to reduce the significant difference in the band power values of each epoch (the value of the features vary between 0 and 1). The control and stress features are combined into one column for every subject, thus creating the total feature matrix. This matrix has a size of 20 × 172. In the next step, labelling will be applied. It helps classify the data between two levels: stressed (1) and not stressed (0). In the total feature matrix, the control phase is labelled as 0, and stress is labelled as 1. Figure 7 provides a summary of the method used to extract the features and classify the two levels of stress. 

### 3.4. Classification of Stress Using EEG Only

**Figure 8:**
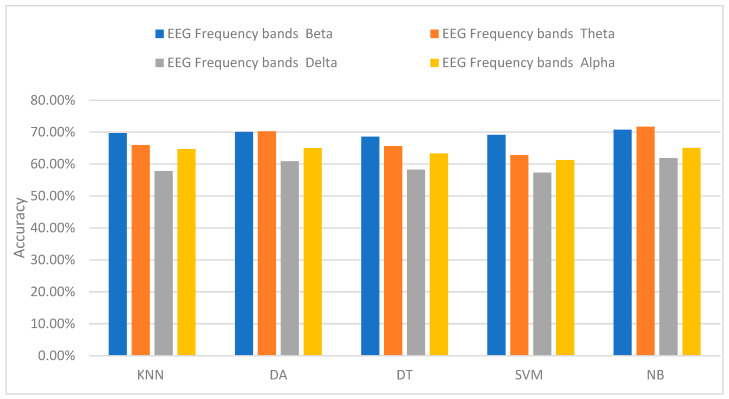
The accuracy of different machine learning classifiers used for every band.

The accuracy of the different classification methods for all of the 20 subjects is shown in Table 1. The classification was implemented on each frequency band of the EEG to determine which band shows a higher level of activity of physiological changes in the EEG. From Table 1, it can be shown that there are differences in the theta and beta bands of almost every subject. The highest accuracy achieved across all frequency bands of the EEG is 70.7% for beta. Figure 8 shows a summary of the different classifiers used for every band. Ultimately, naïve Bayes (NB) provides the highest accuracy amongst the classifiers for all frequency bands. Linear discriminant analysis (LDA) also shows similar results to NB. 

### 3.5. Optimum Electrode Location to Detect Stress Response in EEG

**Figure 9:**
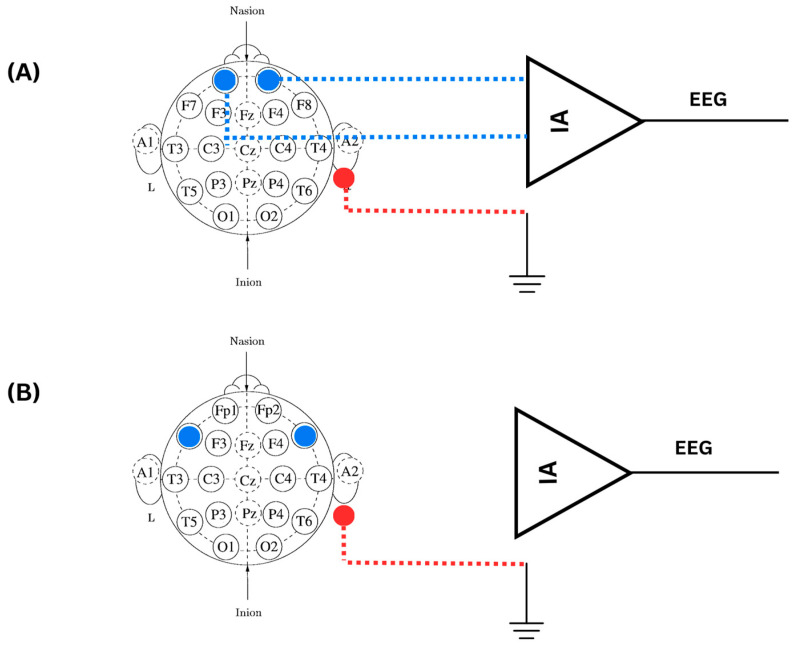
(**A**) The electrode position placed at Fp1 and Fp2. (**B**) The electrode position placed at F7 and Fp8.

The optimum electrode location was investigated at two positions: Fp1 and Fp2 (the prefrontal region) and F8 and F7 (the ventrolateral PFC region), through a statistical analysis. The positions are shown in Figure 9A,B. Ten subjects out of the twenty had electrodes positioned at Fp1 and Fp2. The other ten had electrodes at F8 and F7. The investigation was conducted using the *t*-test tool to examine their significance at a *p*-value less than 0.05. The results of the *p*-value for the twenty subjects are shown in Table 2. 

**Figure 10:**
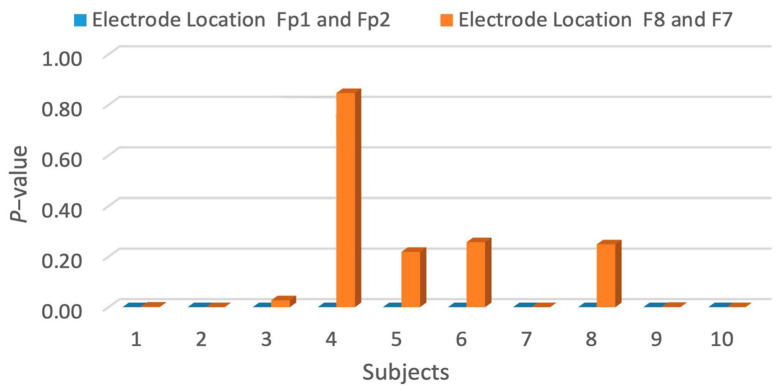
*p*-value at each designated position (Fp1 and Fp2/F7 and F8) of the electrodes during the experiment.

The *p*-values were <0.005 for Fp1 and Fp2. As for F8 and F7, the *p*-value is >0.05 or insignificant for some subjects. The decrease in the *p*-value of the second position could be due to the subjects having more hair and a higher humidity at F8 and F7 than at Fp1 and Fp2. Figure 10 shows a significant difference in term of the *p*-value at each designated position of the electrode during the experiment. The results are on par with the literature review, which states that the prefrontal region is most sensitive to stress [10,11,12]. Nevertheless, the prefrontal region is a position that is sensitive to electrooculograms (EOGs) and blinks. Therefore, the removal of such artifacts is vital for the further assessment of stress.

### 3.6. Subjective and Behavioural Data for Control and Stress Phase

**Figure 11:**
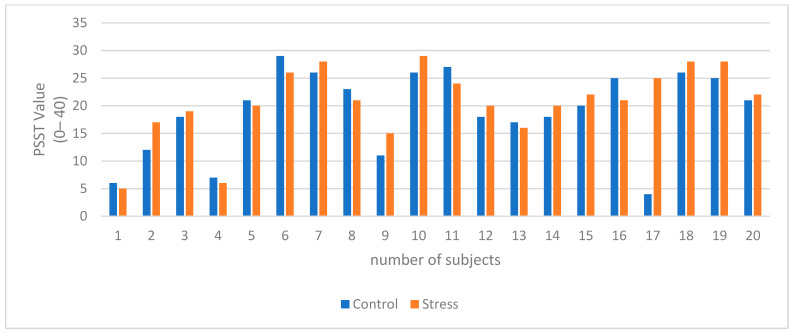
Subjective data of control and stress phases during experiments.

**Figure 12:**
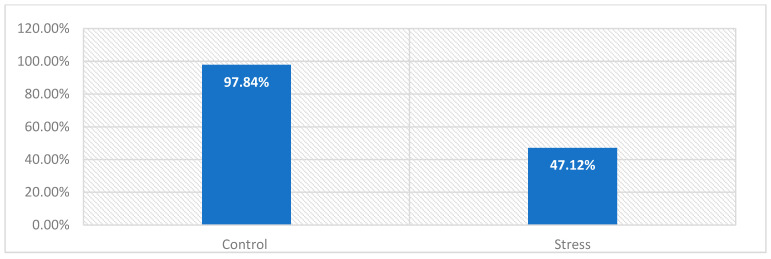
Behavioral accuracy for control and stress phases when participants took the SCWT test.

The perceived stress scale test (PSST) measures the degree to which situations in each subject’s life are perceived as stressful. The questions are intended to assess how unpredictable, uncontrollable, and overloaded the subjects have felt about their life for the past four weeks. The test scores range from 0 to 40. It is divided into three subscales of stress: 0–13 for a low level of stress (LS), 14–26 for a moderate level of stress (MS), and 27–40 for a high level of perceived stress (HS). The PSST is widely used as a reliable measure for physiological stress estimation. Overall, during the experiment, there is an escalation in the PSST score across all twenty subjects from the control phase to the stress phase. Even if they were still in the same stress subscale, the points tend to increase when the control and stress phases are compared in Table 3 and Figure 11. Based on this information, the PSST gives evidence regarding the ability of the SCWT to cause an elevation in stress in subjects during the experiment. In addition, behavioral data suggest that the mean of accuracies for all of the subjects during the control phase was 97.83%. During the stress phase, the accuracy dropped to 47.121%, as shown in Figure 12. The SCWT can induce stress in the participating subjects.

### 3.7. Stress Detection through the Integration of EEGs and GSR

Stress detection through machine learning was achieved using two physiological signals, EEGs and GSR. As mentioned above, band power features were used for the EEGs. However, for GSR, statistical features were used. The total feature matrix will consist of columns indicating the EEG and GSR features of the control and stress phases. The total feature matrix was constructed for the theta and beta bands of the EEG since they indicate significant changes between the control and stress phases. When implementing the total feature matrix, the GSR was also separated into epochs that were equal to those of the EEG to avoid sample size problems when constructing the total feature matrix. The total matrix has a size of 20 × 344 at every frequency of the GSR. Furthermore, the features were normalized to avoid significant differences in the EEG and GSR features. Ultimately, a conclusion on the best frequency and best classification method for EEGs and GSR will be given in this paper. 

Table 4 shows the overall accuracy of fusing GSR and EEGs for KNN, SVM, and NB in comparison to using EEGs alone at the beta and theta bands for all carrier frequencies. It is observed that there is an increase in the accuracy by 14% when comparing using EEGs only with using EEGs and GSR. This resulted from applying the GSR at a low frequency of 100 Hz. At 1 kHz, an increase of 2–10% in accuracy in classifying stress was detected. Ultimately, for the highest carrier frequency, an increase of 13% in classifying stress is established. To conclude, the most successful combination for detecting stress was achieved using the theta band for the EEGs, a 10 kHz frequency for the GSR, and the KNN classifier.

## 4. Discussion

Stress affects the functional connectivity of different brain regions, which can be measured through EEGs. For instance, the frontal cortex, specifically the prefrontal cortex, plays a crucial role in emotional regulation. Stress can impair the prefrontal cortex’s ability to modulate emotions, thus leading to changes in the connectivity pattern of the brain. This disruption will alter the EEG signal. 

Regarding hemispheric asymmetry, stress can induce changes in the laterization of brain activity. Increased right hemisphere activity has been associated with negative emotions and mental stress, while the left hemisphere is involved with positive emotions. EEG studies observe these asymmetrical patterns during stress responses. 

Moreover, this section discusses recommendations on the type of classifier to use and the recommended frequency to use for the acquisition of GSR through the application of ac signals. Furthermore, it will highlight some of the limitations of this system. Most of the classification methods for the EEGs between the stress and control phases obtained the same result for every EEG band. However, naïve Bayes showed the highest accuracy and performance in comparison to the other classification algorithms. In addition, LDA is considered a suitable algorithm for the detection of elevation in stress for the built monitoring system. Overall, NB and LDA showed a high accuracy for the beta band. It can be concluded through the classification of EEG band powers that beta and theta are the most affected by stress. It was reported in previous studies that there is an increase in the activity of the theta band when a subject is in a stress state. The results seen in this paper compliment such observations. 

To improve accuracy, GSR features are added to the total feature matrix of the control and stress phases at three frequencies, namely, 100 Hz, 1 kHz, and 10 kHz. In previous GSR studies, ac current frequencies were set at either a low frequency or a very high frequency. Few studies have used a medium-range frequency for a GSR stimulus. Based on the results, a carrier frequency of 1 kHz led to the lowest increase in accuracy out of the other three. This could be due to the ac method for the acquisition of GSR. In the ac method, the capacitive properties of the skin add to the dc conductance values, resulting in inaccurate conductance readings. The skin capacitance contribution is proportional to the measuring frequency. By using a low measuring frequency, the skin capacitance contribution is reduced to negligible values. Moreover, in comparison to 1 kHz, measurements at higher frequencies used for the estimation of low-frequency conductance preserve responses. Thus, the highest carrier GSR frequency performed well. Ultimately, the chosen frequency will be a trade-off between the ability of the measuring system to detect quick changes and its sensitivity for acquiring sweat duct activity. 

The low-frequency GSR shows the highest accuracy when using the KNN and SVM algorithms among the investigated frequencies. The highest accuracy for GSR classification among the KNN and SVM algorithms was achieved at a frequency of 10 kHz, with the best result being 83.83% in the beta band. Notably, there was a significant increase in the accuracy of the KNN algorithm in the theta band at both the lowest and highest frequencies tested. Therefore, this paper recommends using KNN and SVM classifiers for GSR at 10 kHz and the NB classifier for the EEGs alone to effectively detect elevated stress levels in alignment with the designed mental stress state monitoring system. Table 5 provides a comparison between the proposed device and others in terms of the physiological sensors used, the number of electrodes, and the accuracy of stress detection. When discussing EEG devices for stress detection, it is crucial to consider both standalone EEG systems and those integrated with other physiological signals. The EEG alone offers valuable insights into brain activity patterns associated with stress, but integrating it with additional signals such as heart rate variability (HRV), electrocardiograms (ECGs), and SC can significantly enhance its accuracy. 

The integration of EEGs with other physiological signals provides a more comprehensive understanding of the body’s response to stress. For example, combining EEGs with HRV allows for the simultaneous monitoring of brain activity and cardiovascular function, offering complementary information about the body’s physiological state during stress. Similarly, incorporating ECG and EDA signals provides insights into heart rate changes and skin conductance responses, further enriching the stress detection process.

By integrating EEGs with other physiological signals, the wearability of the device is improved, as it enables a more compact and streamlined design. Users can benefit from a single wearable device capable of monitoring multiple physiological parameters simultaneously, enhancing user comfort and convenience. Moreover, the integration of multiple signals into a single device reduces the overall cost compared to using separate monitoring systems for each parameter.

Furthermore, the combination of EEGs with other physiological signals has been shown to improve the accuracy of stress detection systems. By leveraging complementary information from different modalities, such as brain activity, heart rate, and skin conductance, these integrated systems can achieve a higher accuracy in identifying stress-related patterns. This enhanced accuracy not only improves the reliability of stress detection but also enhances the overall effectiveness of wearable devices for monitoring and managing stress in real-world settings. In order to perceive this concept properly, a comparison between EEG devices is implemented. Several single-channel EEG devices have emerged for various applications, including mental stress monitoring and cognitive assessments. The NeuroSky MindWave uses a single dry electrode on the forehead (FP1) and a reference electrode clipped to the ear, with basic filtering for real-time raw EEG data. It is primarily used for consumer applications like attention training and relaxation, with limited clinical validation but broad adoption due to its ease of use and affordability. The Muse Headband, on the other hand, employs multiple electrodes at FP1, FP2, AF7, and AF8 but typically reports data as a single channel. It integrates advanced algorithms for noise reduction and feature extraction, focusing on meditation and mindfulness training with demonstrated reliability in research and clinical settings. 

Moreover, the Emotiv Insight features five electrodes (AF3, AF4, T7, T8, and Pz) and uses sophisticated signal processing and machine learning algorithms for emotion and cognitive state detection, offering a high accuracy and a broad range of applications, including cognitive performance and mental health assessments. This device has been used in studies as a single channel, utilizing the frontal lobe of the brain to measure different cognitive functions. 

In contrast, our single-channel EEG device measures two physiological signals simultaneously through one channel, which enhances stress analysis while maintaining comfort and compactness. It uses FP1 and FP2 as a differential pair with a common reference, combining differential amplification with band-pass filtering and machine learning for stress detection. This sensor fusion approach provides a significant advantage by improving the accuracy and reliability of stress measurement while keeping the device simple and wearable. Compared to the NeuroSky MindWave and Muse Headband, our device offers a more focused approach to stress monitoring with enhanced signal quality and reliability. Although the Emotiv Insight provides broader cognitive assessments, our device’s specialization ensures effective stress detection with minimal hardware complexity. Our future work will involve extensive clinical validation and the expansion of the device’s application to other cognitive and emotional assessments.

In summary, integrating EEGs with other physiological signals offers several benefits, including improved wearability, reduced cost, and enhanced accuracy in stress detection. These advancements pave the way for the development of more effective and user-friendly wearable devices for stress monitoring and management. Table 5 shows a comparison between the proposed device in this paper and other devices available in the literature.

## 5. Conclusions

In this paper, a stress detection system was designed and tested on hardware and software simulations using Multisim 14.3 and LabVIEW. The system detects two physiological signals through a one-channel sensor. The sensor integrates information from EEGs and GSR when the SNS undergoes physiological changes while performing a mental stress task. Moreover, this system is a wearable monitoring system. Therefore, it is easy to use, portable, low-cost, small, accurate, and can work outside the lab environment for the detection of stress in the workplace. Using EEGs alone resulted in an accuracy of about 70% using the NB classification. However, with GSR, the accuracy was improved to about 80% using KNN and SVM classifiers. 

In GSR studies, an ac excitation current is applied as a stimulus at either high or low frequencies. This signal will carry information related to the electrical changes of the skin. This paper investigates three different levels of frequencies for the classification of stress with EEGs. This paper recommends using a low-frequency GSR at 100 Hz since it provides the highest accuracy. 

When a single channel is used for acquiring an EEG, the accuracy might not be high. Current EEG systems with a high accuracy are high in density and are meant to be used in research labs and clinical settings. Therefore, exploring the optimum location of the single-channel EEG that corresponds to a high sensitivity to stress is crucial. This paper explores two positions: the frontal lobe (F7 and F8) and the prefrontal lobe (Fp1 and Fp2). The results showed that the *p*-value for the prefrontal lobe was much more significant than that for the frontal lobe. 

As a future work, the PCB can be optimized to integrate even more physiological information for further accuracy in the detection of elevated stress. Furthermore, a wireless data acquisition system can be built to speed up the classification of stress and make the system more portable.

## Figures and Tables

**Table 1:** Classification accuracy and sensitivity for EEG band powers.

Accuracy and Sensitivity (Sens.) for Each Classifier (%)	EEG Frequency Bands			
	Beta	Theta	Delta	Alpha
KNN	69.73% ± 0.125 Sens. 64.45%	65.93% ± 0.079 Sens. 59.02%	57.84% ± 0.093 Sens. 56.86%	64.7% ± 0.11 Sens. 57.59%
LDA	70.32% ± 0.134 Sens. 65.75%	64.25% ± 0.082 Sens. 63.35%	60.86% ± 0.091 Sens. 60.69%	65.04% ± 0.11 Sens. 63.95%
DT	68.57% ± 0.121 Sens. 63.38%	65.63% ± 0.074 Sens. 61.15%	58.25% ± 0.089 Sens. 54.78%	63.33% ± 0.13 Sens. 63.1%
SVM	69.16% ± 0.123 Sens. 63.99%	62.85% ± 0.065 Sens. 60.93%	57.29% ± 0.085 Sens. 53.48%	61.23% ± 0.09 Sens. 60.465%
NB	70.74% ± 0.105 Sens. 68.60%	67.7% ± 0.078 Sens. 67.44%	61.86% ± 0.091 Sens. 58.13%	65.07% ± 0.01 Sens. 61.63%

**Table 2:** The *p*-value of investigated electrode location for optimum detection of stress.

*p*-Value for Each Electrode Location			
Subjects		Subjects	
#	Fp1 and Fp2	#	F8 and F7
1	2.22 × 10^−10^	11	1.85 × 10^−3^
2	4.16 × 10^−18^	12	3.44 × 10^−5^
3	5.97 × 10^−13^	13	2.73 × 10^−2^
4	9.42 × 10^−17^	14	8.47 × 10^−1^
5	8.29 × 10^−42^	15	2.19 × 10^−1^
6	1.09 × 10^−6^	16	2.57 × 10^−1^
7	2.56 × 10^−13^	17	4.67 × 10^−5^
8	1.90 × 10^−54^	18	2.48 × 10^−1^
9	1.16 × 10^−10^	19	1.25 × 10^−3^
10	6.67 × 10^−5^	20	9.37 × 10^−5^

**Table 3:** Subjective data in control and stress phases for 20 subjects.

Subjects	Control	Stress	Subjects	Control	Stress
1	6 (LS)	5(LS)	11	27 (HS)	24 (MS)
2	12 (LS)	17 (MS)	12	13 (LS)	20 (MS)
3	18 (MS)	19 (MS)	13	17 (MS)	20(MS)
4	7 (LS)	6 (LS)	14	18 (MS)	20 (MS)
5	21 (MS)	24 (MS)	15	20 (MS)	22 (MS)
6	26 (MS)	28 (HS)	16	25 (MS)	27 (MS)
7	26 (MS)	28 (HS)	17	4 (LS)	25 (MS)
8	23 (MS)	25 (MS)	18	26 (MS)	28 (HS)
9	11 (LS)	15 (MS)	19	25 (MS)	28 (HS)
10	26 (MS)	29 (HS)	20	21 (MS)	22 (MS)

**Table 4:** Accuracy/sensitivity of EEG and GSR at 100 Hz, 1 kHz, and 10 kHz.

Accuracy/Sensitivity of EEG + GSR		
	Accuracy/Sensitivity at Beta band of EEG	
Classification type	EEG + GSR (100 Hz)	EEG
KNN	Accuracy: 83.77%, Sensitivity: 81.16%	Accuracy: 69.73%
DA	Accuracy: 43.39%, Sensitivity: 41.57%	Accuracy: 70.32%
DT	Accuracy: 67.38%, Sensitivity: 62.85%	Accuracy: 68.57%
SVM	Accuracy: 80.04%, Sensitivity: 79.05%	Accuracy: 69.16%
NB	Accuracy: 72.58%, Sensitivity: 73.52%	Accuracy: 70.74%
Accuracy at Theta band of EEG		
Classification type	EEG + GSR (100 Hz)	EEG
KNN	Accuracy: 79.58%, Sensitivity:78.97%	Accuracy: 69.73%
DA	Accuracy: 52.06%, Sensitivity: 54.27%	Accuracy: 70.32%
DT	Accuracy: 58.85%, Sensitivity: 57.31%	Accuracy: 68.57%
SVM	Accuracy: 73.57%, Sensitivity:73.37%	Accuracy: 69.16%
NB	Accuracy: 76.04%, Sensitivity: 77.2%	Accuracy: 70.74%
Accuracy at Beta band of EEG		
Classification type	EEG + GSR (1 kHz)	EEG
KNN	Accuracy: 75.44%, Sensitivity: 73.89%	Accuracy: 69.73%
DA	Accuracy: 42.77%, Sensitivity: 48.82%	Accuracy: 70.32%
DT	Accuracy: 58.70%, Sensitivity: 60.92%	Accuracy: 68.57%
SVM	Accuracy: 74.46%, Sensitivity: 72.19%	Accuracy: 69.16%
NB	Accuracy: 72.56%, Sensitivity: 71.63%	Accuracy: 70.74%
Accuracy at Theta band of EEG		
Classification type	EEG + GSR (1 kHz)	EEG
KNN	Accuracy: 73.33%, Sensitivity: 68.64%	Accuracy: 65.93%
DA	Accuracy: 56.23%, Sensitivity: 58.79%	Accuracy: 64.25%
DT	Accuracy: 55.91%, Sensitivity: 56.72%	Accuracy: 65.62%
SVM	Accuracy: 69.63%, Sensitivity: 67.52%	Accuracy: 62.85%
NB	Accuracy: 73.71%, Sensitivity: 77.56%	Accuracy: 67.70%
Accuracy at Beta band for EEG		
Classification type	EEG + GSR (10 kHz)	EEG
KNN	Accuracy: 83.44%, Sensitivity: 84.22%	Accuracy: 69.73%
DA	Accuracy: 42.89%, Sensitivity: 41.90%	Accuracy: 70.32%
DT	Accuracy: 43.06%, Sensitivity: 42.36%	Accuracy: 68.57%
SVM	Accuracy: 79.25%, Sensitivity: 74.44%	Accuracy: 69.16%
NB	Accuracy: 64.52%, Sensitivity: 62.78%	Accuracy: 70.74%
Accuracy at Theta band for EEG		
Classification type	EEG + GSR (10 kHz)	EEG
KNN	Accuracy: 83.83%, Sensitivity: 82.90%	Accuracy: 69.73%
DA	Accuracy: 45.55%, Sensitivity: 43.73%	Accuracy: 70.32%
DT	Accuracy: 58.74%, Sensitivity: 54.27%	Accuracy: 68.57%
SVM	Accuracy: 77.98%, Sensitivity: 73.53%	Accuracy: 69.16%
NB	Accuracy: 64.55%, Sensitivity: 62.39%	Accuracy: 70.74%
Accuracy/Sensitivity at Beta band of EEG		
Classification type	EEG + GSR (100 Hz)	EEG
KNN	Accuracy: 83.77%, Sensitivity: 81.16%	Accuracy: 69.73%
DA	Accuracy: 43.39%, Sensitivity: 41.57%	Accuracy: 70.32%
DT	Accuracy: 67.38%, Sensitivity: 62.85%	Accuracy: 68.57%
SVM	Accuracy: 80.04%, Sensitivity: 79.05%	Accuracy: 69.16%
NB	Accuracy: 72.58%, Sensitivity: 73.52%	Accuracy: 70.74%
Accuracy at Theta band of EEG		
Classification type	EEG + GSR (100 Hz)	EEG
KNN	Accuracy: 79.58%, Sensitivity:78.97%	Accuracy: 69.73%
DA	Accuracy: 52.06%, Sensitivity: 54.27%	Accuracy: 70.32%
DT	Accuracy: 58.85%, Sensitivity: 57.31%	Accuracy: 68.57%
SVM	Accuracy: 73.57%, Sensitivity:73.37%	Accuracy: 69.16%
NB	Accuracy: 76.04%, Sensitivity: 77.2%	Accuracy: 70.74%
Accuracy at Beta band of EEG		
Classification type	EEG + GSR (1 kHz)	EEG
KNN	Accuracy: 75.44%, Sensitivity: 73.89%	Accuracy: 69.73%
DA	Accuracy: 42.77%, Sensitivity: 48.82%	Accuracy: 70.32%
DT	Accuracy: 58.70%, Sensitivity: 60.92%	Accuracy: 68.57%
SVM	Accuracy: 74.46%, Sensitivity: 72.19%	Accuracy: 69.16%
NB	Accuracy: 72.56%, Sensitivity: 71.63%	Accuracy: 70.74%
Accuracy at Theta band of EEG		
Classification type	EEG + GSR (1 kHz)	EEG
KNN	Accuracy: 73.33%, Sensitivity: 68.64%	Accuracy: 65.93%
DA	Accuracy: 56.23%, Sensitivity: 58.79%	Accuracy: 64.25%
DT	Accuracy: 55.91%, Sensitivity: 56.72%	Accuracy: 65.62%
SVM	Accuracy: 69.63%, Sensitivity: 67.52%	Accuracy: 62.85%
NB	Accuracy: 73.71%, Sensitivity: 77.56%	Accuracy: 67.70%
Accuracy at Beta band for EEG		
Classification type	EEG + GSR (10 kHz)	EEG
KNN	Accuracy: 83.44%, Sensitivity: 84.22%	Accuracy: 69.73%
DA	Accuracy: 42.89%, Sensitivity: 41.90%	Accuracy: 70.32%
DT	Accuracy: 43.06%, Sensitivity: 42.36%	Accuracy: 68.57%
SVM	Accuracy: 79.25%, Sensitivity: 74.44%	Accuracy: 69.16%
NB	Accuracy: 64.52%, Sensitivity: 62.78%	Accuracy: 70.74%
Accuracy at Theta band for EEG		
Classification type	EEG + GSR (10 kHz)	EEG
KNN	Accuracy: 83.83%, Sensitivity: 82.90%	Accuracy: 69.73%
DA	Accuracy: 45.55%, Sensitivity: 43.73%	Accuracy: 70.32%
DT	Accuracy: 58.74%, Sensitivity: 54.27%	Accuracy: 68.57%
SVM	Accuracy: 77.98%, Sensitivity: 73.53%	Accuracy: 69.16%
NB	Accuracy: 64.55%, Sensitivity: 62.39%	Accuracy: 70.74%

**Table 5:** Comparison between different studies in the literature review.

Study	Number of Channels	Type of Stressor	Electrode Location on Brain Region	Methodology	Accuracy
[57]	14	SCWT	Frontal, Temporal, Occipital	SCWT was used in 18 patients to elicit stress. Each session consisted of 24 trials; each lasted for 1 s. Logistic regression, KNN, and QDA were developed to obtain the accuracy.	Accuracy: 70.71% Alpha waves are significant at the pre-frontal lobe
[58]	10	SCWT, mental arithmetic	Frontal, Central, Temporal, Parietal, Occipital	Twelve subjects took the SCWT. They rested for 60 s (control) and then took the SCWT twice for 30 s. They used PSD for feature extraction and logistic regression. XGBoost, SVM, DT, and random forest were used for classification of mental stress.	Accuracy is 86.49%
[59]	14	SCWT	Frontal, Temporal, occipital	The SCWT was designed to wait only one second to obtain the user’s response. This was performed for 10 sessions in order to induce mental stress. Logistic regression and KNN were used to further classify stress.	Accuracy: 73.96%
[45]	30	SCWT	Right prefrontal region	Highest sensitivity to stress was at the right pre-frontal lobe region. High accuracy due to the combination of EEG with FNIRs and stress mitigation was found.	Accuracy of EEG increased by 20.83%
This paper	1	SCWT	Fp1 and FP2 (prefrontal region)	Through sensor fusion, EEG and GSR were used to classify stress. This improved overall accuracy.	Accuracy: 83.7%

## Data Availability

The data can be obtained from the corresponding author following our lab’s protocol for data sharing.

## Appendix A

**Table A1:** Acronyms, full names, and descriptions in this manuscript.

Acronym	Full Name	Description
EEG	Electroencephalogram	A method used to record the electrical activity of the brain, primarily used in this context for monitoring mental stress.
GSR	Galvanic Skin Response	A method of measuring the electrical conductance of the skin, which varies with its moisture level due to sweat, used as an indicator of psychological or physiological arousal.
SCWT	Stroop Color and Word Test	A psychological test involving the naming of the color of a word which is printed in a different color and that is used to induce stress and measure cognitive function.
SNS	Sympathetic Nervous System	Part of the autonomic nervous system that activates the fight-or-flight response, releasing stress hormones.
ANS	Autonomic Nervous System	Controls involuntary bodily functions such as heart rate, digestion, and respiratory rate and is involved in stress responses.
CNS	Central Nervous System	Comprises the brain and spinal cord and controls most functions of the body and mind.
RESPR	Respiration Rate	The number of breaths taken per minute, used in monitoring physiological responses to stress.
ECG	Electrocardiogram	A recording of the electrical activity of the heart, sometimes used in stress monitoring.
SC	Skin Conductance	Another term for galvanic skin response, measuring the conductance of the skin which varies with moisture level.
EDA	Electrodermal Activity	A measure of the skin’s ability to conduct electricity, which varies with its moisture level and is used to gauge emotional and physiological arousal.
TSST	Trier Social Stress Test	A commonly used protocol to induce stress in research participants through public speaking and mental arithmetic tasks.
FP1	Frontopolar 1	An electrode position in the EEG system, located at the front of the scalp.
FP2	Frontopolar 2	An electrode position in the EEG system, located at the front of the scalp.
F7	Frontal 7	An electrode position in the EEG system, located at the front of the scalp, on the left side.
F8	Frontal 8	An electrode position in the EEG system, located at the front of the scalp, on the right side.
SLS	Selective Laser Sintering	A three-dimensional printing technology that uses a laser to sinter powdered material, binding it together to create a solid structure, often used for creating prototypes and end-use parts.
PA12	Polyamide 12	A type of thermoplastic polymer used in three-dimensional printing and other applications.
PSS	Perceived Stress Scale	A psychological instrument used to measure the perception of stress.
PSD	Power Spectral Density	A measure of the power present in a signal as a function of frequency, used in signal processing.
IRB	Institutional Review Board	A committee that reviews and approves research involving human subjects to ensure ethical standards are met.
ADC	Analog-to-Digital Converter	A device that converts analog signals to digital data for processing.
KNN	K-Nearest Neighbors	A machine learning algorithm used for classification and regression tasks.
SVM	Support Vector Machine	A supervised learning algorithm used for classification and regression analysis.
DT	Decision Tree	A decision support tool that uses a tree-like model of decisions and their possible consequences.
DA	Discriminant Analysis	A statistical technique used to classify a set of observations into predefined classes.
NB	Naive Bayes	A classification technique based on Bayes’s theorem with an assumption of independence between predictors.
LDA	Linear Discriminant Analysis	A classification and dimensionality reduction technique in machine learning.
V-I	Voltage–Current	Refers to the relationship between voltage and current in electrical circuits.
EMG	Electromyography	A technique for evaluating and recording the electrical activity produced by skeletal muscles.
EOG	Electrooculography	A technique for measuring the corneo-retinal standing potential that exists between the front and the back of the human eye.
LS	Low Stress	A category indicating a low level of stress, determined by the perceived stress scale.
MS	Medium Stress	A category indicating a medium level of stress, determined by the perceived stress scale.
HS	High Stress	A category indicating a high level of stress, determined by the perceived stress scale.
HRV	Heart Rate Variability	The variation in the time interval between heartbeats, used as an indicator of stress and autonomic nervous system activity.
Sens.	Sensitivity	Measures how well a machine learning model can detect positive instances.

## References

[1] Lupien S.J., McEwen B.S., Gunnar M.R., Heim C. (2009). Effects of stress throughout the lifespan on the brain, behaviour and cognition. Nat. Rev. Neurosci..

[2] McEwen B.S. (2007). Physiology and neurobiology of stress and adaptation: Central role of the brain. Physiol. Rev..

[3] Masri G., Al-Shargie F., Tariq U., Almughairbi F., Babiloni F., Al-Nashash H. (2023). Mental Stress Assessment in the Workplace: A Review. IEEE Trans. Affect. Comput..

[4] Selye H. (1950). Stress and the general adaptation syndrome. Br. Med. J..

[5] Giannakakis G., Grigoriadis D., Giannakaki K., Simantiraki O., Roniotis A., Tsiknakis M. (2019). Review on psychological stress detection using biosignals. IEEE Trans. Affect. Comput..

[6] Carneiro D., Novais P., Augusto J.C., Payne N. (2017). New methods for stress assessment and monitoring at the workplace. IEEE Trans. Affect. Comput..

[7] Ossewaarde L., Qin S., Van Marle H.J.F., van Wingen G.A., Fernández G., Hermans E.J. (2011). Stress-induced reduction in reward-related prefrontal cortex function. Neuroimage.

[8] Vanhollebeke G., Kappen M., De Raedt R., Baeken C., van Mierlo P., Vanderhasselt M.-A. (2023). Effects of acute psychosocial stress on source level EEG power and functional connectivity measures. Sci. Rep..

[9] Vanhollebeke G., De Smet S., De Raedt R., Baeken C., van Mierlo P., Vanderhasselt M.-A. (2022). The neural correlates of psychosocial stress: A systematic review and meta-analysis of spectral analysis EEG studies. Neurobiol. Stress.

[10] Katmah R., Al-Shargie F., Tariq U., Babiloni F., Al-Mughairbi F., Al-Nashash H. (2021). A review on mental stress assessment methods using EEG signals. Sensors.

[11] Al-Shargie F.M., Tang T.B., Badruddin N., Kiguchi M. (2016). Mental stress quantification using EEG signals. Proceedings of the International Conference for Innovation in Biomedical Engineering and Life Sciences: ICIBEL2015.

[12] Attallah O. (2020). An Effective Mental Stress State Detection and Evaluation System Using Minimum Number of Frontal Brain Electrodes. Diagnostics.

[13] Hou X., Liu Y., Sourina O., Tan Y.R.E., Wang L., Müller-Wittig W. EEG Based Stress Monitoring. Proceedings of the 2015 IEEE International Conference on Systems, Man, and Cybernetics.

[14] Karthikeyan P., Murugappan M., Yaacob S. A review on stress inducement stimuli for assessing human stress using physiological signals. Proceedings of the 2011 IEEE 7th International Colloquium on Signal Processing and Its Applications.

[15] Jun G., Smitha K.G. EEG based stress level identification. Proceedings of the 2016 IEEE International Conference on Systems, Man, and Cybernetics (SMC).

[16] Shanmugasundaram G., Yazhini S., Hemapratha E., Nithya S. A comprehensive review on stress detection techniques. Proceedings of the 2019 IEEE International Conference on System, Computation, Automation and Networking (ICSCAN).

[17] Cantara A., Ceniza A. (2016). Stress Sensor Prototype: Determining the Stress Level in using a Computer through Validated Self-Made Heart Rate (HR) and Galvanic Skin Response (GSR) Sensors and Fuzzy Logic Algorithm. Int. J. Eng. Res. Technol..

[18] Grimnes S., Jabbari A., Martinsen Ø.G., Tronstad C. (2011). Electrodermal activity by DC potential and AC conductance measured simultaneously at the same skin site. Skin Res. Technol..

[19] Nordbotten B.J., Tronstad C., Martinsen Ø.G., Grimnes S. (2014). Estimation of skin conductance at low frequencies using measurements at higher frequencies for EDA applications. Physiol. Meas..

[20] Das P., Das A., Tibarewala D., Khasnobish A. Design and development of portable galvanic skin response acquisition and analysis system. Proceedings of the 2016 International Conference on Intelligent Control Power and Instrumentation (ICICPI).

[21] Posada-Quintero H.F., Chon K.H. (2020). Innovations in electrodermal activity data collection and signal processing: A systematic review. Sensors.

[22] Kim J., Kwon S., Seo S., Park K. Highly wearable galvanic skin response sensor using flexible and conductive polymer foam. Proceedings of the 2014 36th Annual International Conference of the IEEE Engineering in Medicine and Biology Society.

[23] Banganho A., Santos M., da Silva H.P. (2021). Design and Evaluation of an Electrodermal Activity Sensor (EDA) With Adaptive Gain. IEEE Sens. J..

[24] Sequeira H., Roy J.-C. (1993). Cortical and hypothalamo-limbic control of electrodermal responses. Progress in Electrodermal Research.

[25] Boucsein W. (2012). Principles of electrodermal phenomena. Electrodermal Activity.

[26] Secerbegovic A., Ibric S., Nisic J., Suljanovic N., Mujcic A. (2017). Mental workload vs. stress differentiation using single-channel EEG. CMBEBIH 2017.

[27] Healey J.A., Picard R.W. (2005). Detecting stress during real-world driving tasks using physiological sensors. IEEE Trans. Intell. Transp. Syst..

[28] Panicker S.S., Gayathri P. (2019). A survey of machine learning techniques in physiology based mental stress detection systems. Biocybern. Biomed. Eng..

[29] Fowles D.C. (1993). Electrodermal activity and antisocial behavior: Empirical findings and theoretical issues. Progress in Electrodermal Research.

[30] Anusha A., Jose J., Preejith S., Jayaraj J., Mohanasankar S. (2018). Physiological signal based work stress detection using unobtrusive sensors. Biomed. Phys. Eng. Express.

[31] Tyagi A., Semwal S., Shah G. (2012). A review of EEG sensors used for data acquisition. J. Comput. Appl. (IJCA).

[32] Sano A., Taylor S., McHill A.W., Phillips A.J., Barger L.K., Klerman E., Picard R. (2018). Identifying Objective Physiological Markers and Modifiable Behaviors for Self-Reported Stress and Mental Health Status Using Wearable Sensors and Mobile Phones: Observational Study. J. Med. Internet Res..

[33] Sani M., Norhazman H., Omar H., Zaini N., Ghani S. Support vector machine for classification of stress subjects using EEG signals. Proceedings of the 2014 IEEE Conference on Systems, Process and Control (ICSPC 2014).

[34] Zanetti M., Mizumoto T., Faes L., Fornaser A., De Cecco M., Maule L., Valente M., Nollo G. (2019). Multilevel assessment of mental stress via network physiology paradigm using consumer wearable devices. J. Ambient. Intell. Humaniz. Comput..

[35] Ahn J.W., Ku Y., Kim H.C. (2019). A Novel Wearable EEG and ECG Recording System for Stress Assessment. Sensors.

[36] Alberdi A., Aztiria A., Basarab A. (2016). Towards an automatic early stress recognition system for office environments based on multimodal measurements: A review. J. Biomed. Inform..

[37] Seoane F., Mohino-Herranz I., Ferreira J., Alvarez L., Buendia R., Ayllón D., Llerena C., Gil-Pita R. (2014). Wearable biomedical measurement systems for assessment of mental stress of combatants in real time. Sensors.

[38] Baumgartl H., Fezer E., Buettner R. Two-Level Classification of Chronic Stress in EEG Recordings Two-Level Classification of Chronic Stress Using Machine Learning on Resting-State EEG Recordings. Proceedings of the AMCIS 2020 Proceedings: 25th Americas Conference on Information Systems.

[39] Scarpina F., Tagini S. (2017). The Stroop Color and Word Test. Front. Psychol..

[40] Bousefsaf F., Maaoui C., Pruski A. Remote assessment of the Heart Rate Variability to detect mental stress. Proceedings of the 2013 7th International Conference on Pervasive Computing Technologies for Healthcare and Workshops (PervasiveHealth).

[41] Golden Z.L., Golden C.J. (2002). Patterns of performance on the Stroop Color and Word Test in children with learning, attentional, and psychiatric disabilities. Psychol. Sch..

[42] Derrfuss J., Brass M., Neumann J., Von Cramon D.Y. (2005). Involvement of the inferior frontal junction in cognitive control: Meta-analyses of switching and Stroop studies. Hum. Brain Mapp..

[43] Williams J.M., Mathews A., MacLeod C. (1996). The emotional Stroop task and psychopathology. Psychol. Bull..

[44] Amatachaya S., Srisim K., Arrayawichanon P., Thaweewannakij T., Amatachaya P. (2019). Dual-Task Obstacle Crossing Training Could Immediately Improve Ability to Control a Complex Motor Task and Cognitive Activity in Chronic Ambulatory Individuals With Spinal Cord Injury. Top. Spinal Cord Inj. Rehabil..

[45] Katmah R., Al-Shargie F., Tariq U., Babiloni F., Al-Mughairbi F., Al-Nashash H. (2023). Mental Stress Management Using fNIRS Directed Connectivity and Audio Stimulation. IEEE Trans. Neural Syst. Rehabil. Eng..

[46] Al-Shargie F., Katmah R., Tariq U., Babiloni F., Al-Mughairbi F., Al-Nashash H. (2022). Stress management using fNIRS and binaural beats stimulation. Biomed. Opt. Express.

[47] Yoo J., Park J.W., Kim S.J. Development of User-friendly Bio-signal Acquisition System Based on LabVIEW. Proceedings of the 2016 IEEE International Conference on Consumer Electronics (ICCE).

[48] Chi Y.M., Deiss S.R., Cauwenberghs G. Non-contact low power EEG/ECG electrode for high density wearable biopotential sensor networks. Proceedings of the 2009 Sixth International Workshop on Wearable and Implantable Body Sensor Networks.

[49] Sierra A.D.S., Ávila C.S., Casanova J.G., Bailador G. (2011). A Stress-Detection System Based on Physiological Signals and Fuzzy Logic. IEEE Trans. Ind. Electron..

[50] Cohen S., Kamarck T., Mermelstein R. (1994). Perceived stress scale. Measuring Stress: A Guide for Health and Social Scientists.

[51] Amin A.H.U., Malik A., Ahmad R.F., Badruddin N., Kamel N., Hussain M., Chooi W.-T. (2015). Feature extraction and classification for EEG signals using wavelet transform and machine learning techniques. Australas. Phys. Eng. Sci. Med..

[52] Hou X., Liu Y., Sourina O., Mueller-Wittig W. CogniMeter: EEG-based emotion, mental workload and stress visual monitoring. Proceedings of the 2015 International Conference on Cyberworlds (CW).

[53] Richard N.Y., Benjamin B.G., Brian L.S. (2005). An overview of power spectral density (PSD) calculations. Proc. SPIE.

[54] Al-Shargie F.M., Hassanin O., Tariq U., Al-Nashash H. (2020). EEG-based semantic vigilance level classification using directed connectivity patterns and graph theory analysis. IEEE Access.

[55] Bobade P., Vani M. Stress Detection with Machine Learning and Deep Learning using Multimodal Physiological Data. Proceedings of the 2020 Second International Conference on Inventive Research in Computing Applications (ICIRCA).

[56] Badr Y., Al-Shargie F., Tariq U., Babiloni F., Al-Mughairbi F., Al-Nashash H. Mental Stress Detection and Mitigation using Machine Learning and Binaural Beat Stimulation. Proceedings of the 2023 45th Annual International Conference of the IEEE Engineering in Medicine & Biology Society (EMBC).

[57] Blanco J.A., Vanleer A.C., Calibo T.K., Firebaugh S.L. (2019). Single-Trial Cognitive Stress Classification Using Portable Wireless Electroencephalography. Sensors.

[58] Tsai Y.-H., Wu S.-K., Yu S.-S., Tsai M.-H. (2022). Analyzing Brain Waves of Table Tennis Players with Machine Learning for Stress Classification. Appl. Sci..

[59] Calibo T.K., Blanco J.A., Firebaugh S.L. Cognitive stress recognition. Proceedings of the 2013 IEEE International Instrumentation and Measurement Technology Conference (I2MTC).

